# Interleukin-6-stimulated progranulin expression contributes to the malignancy of hepatocellular carcinoma cells by activating mTOR signaling

**DOI:** 10.1038/srep21260

**Published:** 2016-02-16

**Authors:** Feng Liu, Wen Zhang, Fusheng Yang, Tingting Feng, Meng Zhou, Yuan Yu, Xiuping Yu, Weiming Zhao, Fan Yi, Wei Tang, Yi Lu

**Affiliations:** 1 Department of Biochemistry and Molecular Biology, Shandong University School of Medicine, Jinan 250012, China; 2 Department of Pathogenic Biology, Shandong University School of Medicine, Jinan 250012, China; 3 Department of Pharmacology, Shandong University School of Medicine, Jinan 250012, China; 4 Department of Hematology, Qilu Hospital, Shandong University, Jinan 250012, China; 5 Department of Clinical Laboratory, The Second Affiliated Hospital of Xi’an Jiaotong University, Xi’an 710004, China

## Abstract

This study aimed to determine the expression of progranulin (PGRN) in hepatocellular carcinoma (HCC) cells in response to interleukin 6 (IL-6), a non-cellular component of the tumor microenvironment, and the molecular mechanism of PGRN oncogenic activity in hepatocarcinogenesis. Levels of IL-6 and PGRN were increased and positively correlated in HCC tissues. IL-6 dose- and time-dependently increased PGRN level in HCC cells. IL-6-driven PGRN expression was at least in part mediated by Erk/C/EBPβ signaling, and reduced expression of PGRN impaired IL-6-stimulated proliferation, migration and invasion of HepG2 cells. PGRN activated mammalian target of rapamycin (mTOR) signaling, as evidenced by increased phosphorylation of p70S6K, 4E-BP1, and Akt-Ser473/FoxO1. Inhibition of mTOR signaling with rapamycin, an mTOR signaling inhibitor, disturbed PGRN- or IL-6-mediated proliferation, migration and invasion of HCC cells *in vitro*. Persistent activation of mTOR signaling by knockdown of TSC2 restored PGRN-knockdown-attenuated pro-proliferation effects of IL-6 in HepG2 cells. In addition, rapamycin treatment *in vivo* in mice slowed tumor growth stimulated by recombinant human PGRN. Our findings provide a better understanding of the biological activities of the IL-6/PGRN/mTOR cascade in the carcinogenesis of HCC, which may suggest a novel target in the treatment of HCC.

Liver cancer is one of the most common malignant tumors and leading causes of cancer-related deaths worldwide, responsible for an estimated incidence of 782,500 cases and 745,500 deaths during 2012; China alone accounts for about 50% of the total number of cases and deaths^1^. Primary liver cancers include hepatocellular carcinoma (HCC), cholangiocarcinoma, hepatoblastoma, bile duct cystadenocarcinoma and haemangiosarcoma. HCC is the most common, accounting for 85–90% of primary liver cancer cases^2^. Chronic infection with hepatitis B virus (HBV) and HCV, alcohol abuse, and nonalcoholic fatty liver disease are the major risk factors for HCC^3^.

Recent studies have highlighted a requirement for cross-talk between tumor cells and their surrounding microenvironment in HCC development^4^. As a non-cellular component of the microenvironment, interleukin 6 (IL-6) is one of the best-characterized pro-tumorigenic cytokines^5^. The expression of IL-6 is increased in both liver cirrhosis and HCC^6^,^7^ and is associated with rapid progression from viral hepatitis to HCC^8^,^9^.

Progranulin (PGRN), also referred to as granulin-epithelin precursor, is a 593 amino-acid autocrine growth factor containing 7.5 repeats of a cysteine-rich motif and forms a unique “beads-on-a-string” structure^10^. PGRN plays a critical role in various physiological processes and is involved in the pathogenesis of many types of diseases, such as autoimmune disorders, cancer, atherosclerosis, obesity, and neurodegenerative diseases^11^,^12^,^13^,^14^,^15^,^16^.

Elevated PGRN levels often occur in a variety of human cancers, and PGRN is strongly believed to contribute to tumorigenesis^16^,^17^. PGRN can activate the phosphatidylinositol-3-kinase (PI3K) and extracellular signal-regulated kinase (Erk1/2) signaling pathways, required for proliferation, cell survival, and invasion of cancer cells^17^. In addition, PGRN stimulates phosphorylation of the 70S ribosomal protein S6 kinase (p70S6K)^17^,^18^, a downstream target of PI3K/Akt/mammalian target of rapamycin (mTOR) signaling.

The mRNA and protein levels of PGRN were found overexpressed in more than 70% of HCC samples^19^,^20^. *In vitro*, PGRN is involved in cell proliferation, invasion, migration and chemo-resistance of HCC cells^20^,^21^. Increased PGRN levels have been found in tumors and serum of patients with cholangiocarcinoma, the second most common primary cancer of the liver, as compared with healthy controls^22^. IL-6, overexpressed in cholangiocarcinoma, drives the expression of PGRN by activating Erk1/2/RSK1/C/EBPβ signaling^22^. Furthermore, PGRN has growth-promoting effects on cholangiocytes by inactivating forkhead box protein O1 (FoxO1), a downstream target of Akt signaling^22^.

The PI3K/Akt/mTOR signaling pathway plays a crucial role in cell growth, proliferation and survival and is frequently activated in human cancer. mTOR exists in two different complexes, mTORC1 and mTORC2. mTORC1 acts as a key regulator of cell growth and proliferation by regulating protein synthesis and allows progression from the G1 to S phase of the cell cycle^23^. mTORC2 is required for activation of Akt via its phosphorylation of serine 473^24^. The mTOR pathway is upregulated in 40% to 50% of HCC samples, and mTOR inhibitors have shown antitumoral activity in experimental models of HCC^25^,^26^,^27^,^28^.

In the present study, we aimed to determine the expression of PGRN in HCC cells in response to IL-6 and the molecular mechanism of PGRN oncogenic activity in hepatocarcinogenesis. Increased PGRN expression from HCC cells in response to IL-6 stimulation was via MEK/Erk signaling activating the transcriptional factor C/EBPβ. PGRN promoted the activation of mTOR signaling, which was required for PGRN-mediated proliferation, survival, migration and invasion in HCC cells. Furthermore, blocking mTOR signaling slowed the growth of HCC *in vivo*.

## Results

### IL-6 and PGRN levels were positively correlated in HCC tissues and PGRN expression was increased in HCC cells treated with IL-6.

**Figure 1:**
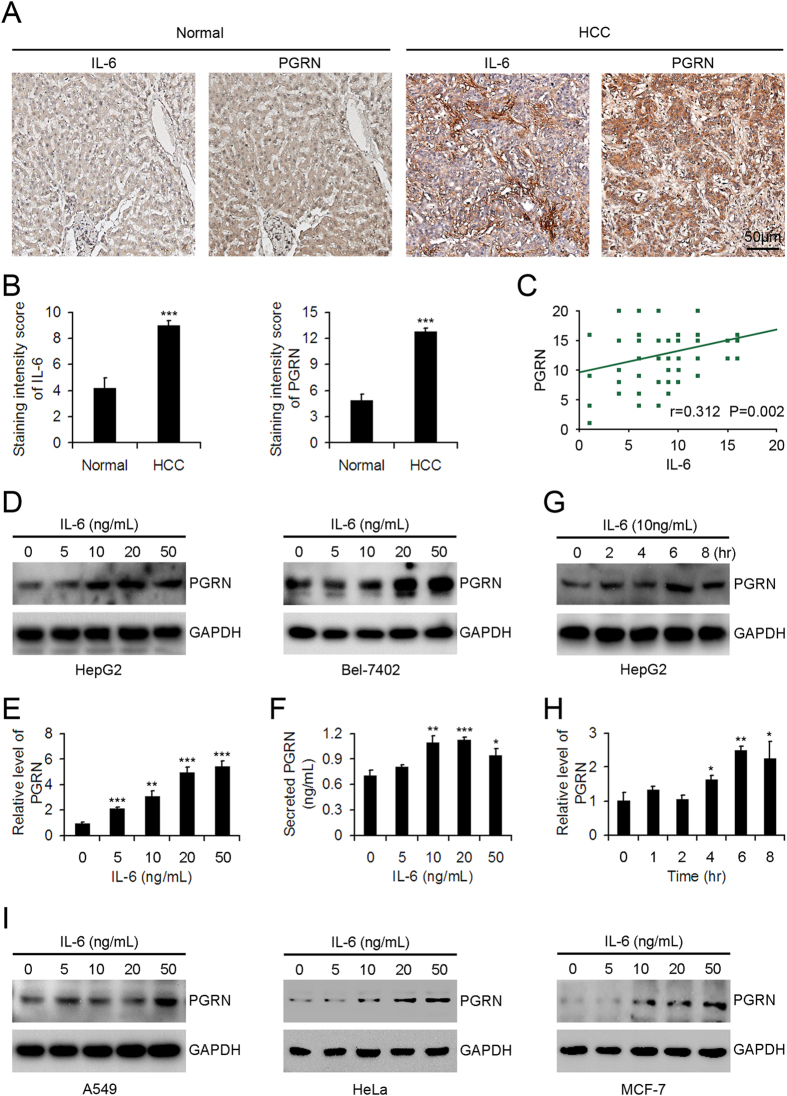
Positive correlation between interleukin 6 (IL-6) and progranulin (PGRN) levels in hepatocellular carcinoma (HCC) tissues and IL-6 elevated the expression of PGRN in HCC cells. (**A**) Representative photomicrographs of IL-6 and PGRN immunohistochemical staining in normal liver and HCC samples. (**B**) Quantification of staining intensity. Data are mean ± SEM. ***p < 0.001 compared with the normal liver tissues. (**C**) Pearson correlation analysis of IL-6 and PGRN expression in HCC. (**D**) Western blot assay of intracellular PGRN protein expression in HepG2 and Bel-7402 cells treated with 0, 5, 10, 20, or 50 ng/mL IL-6 for 6 hr. GAPDH was a loading control. Quantitative PCR (qPCR) analysis (**E**) and ELISA assay (**F**) of PGRN mRNA expression and secreted PGRN levels in HepG2 cells treated with doses of IL-6 for 6 hr. Western blot assay (**G**) and qPCR analysis (**H**) of intracellular PGRN protein and mRNA expression in HepG2 cells treated with 10 ng/mL IL-6 at the indicated times. (**I**) Western blot assay of intracellular PGRN protein expression in A549, HeLa and MCF-7 cells treated with 0, 5, 10, 20, or 50 ng/mL IL-6 for 6 hr. GAPDH was a loading control. Data are mean ± SD. *p < 0.05; **p < 0.01; ***p < 0.001 compared with IL-6, 0 ng/mL or 0 hr.

Immunohistochemistry revealed greater IL-6 and PGRN immunoreactivity in HCC tissues compared with normal liver tissues (Fig. 1A,B). Interestingly, accompanying with the holistic enhancement of IL-6 and PGRN expression in HCC tissues, the elevated IL-6 and PGRN levels in HCC were mainly observed in mesenchymal and epithelia tissues, respectively. Importantly, the levels of IL-6 and PGRN in HCC tissues were positively correlated (Fig. 1C). Treatment with recombinant human IL-6 dose dependently increased the intracellular protein levels of PGRN in HCC HepG2 and Bel-7402 cells (Fig. 1D). Levels of mRNA and secreted PGRN were dose-dependently increased in HepG2 cells stimulated with 0, 5, 10, 20 or 50 ng/mL IL-6 (Fig. 1E,F). Furthermore, the intracellular protein and mRNA levels of PGRN were time-dependently increased in HepG2 cells with 10 ng/mL IL-6 treatment (Fig. 1G,H). In addition, IL-6-driven PGRN expression was also confirmed in lung, cervical and breast cancer A549, HeLa and MCF-7 cell lines.

### IL-6-driven PGRN expression in HepG2 cells depended on Erk/C/EBPβ signaling.

**Figure 2:**
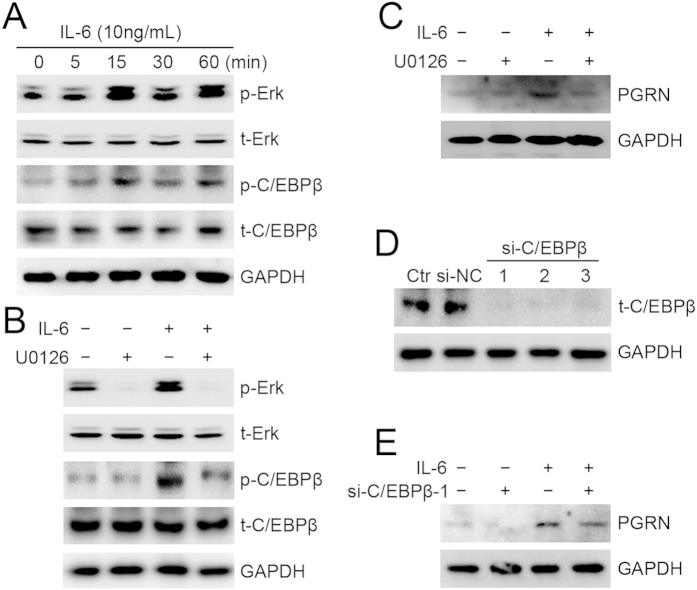
IL-6-stimulated Erk/C/EBPβ signaling contributed to PGRN expression in HepG2 cells. (**A**) Western blot assay of Erk and C/EBPβ activation with 10 ng/mL IL-6 treatment for 0, 5, 15, 30, and 60 min in HepG2 cells. (**B**) Activation of Erk and C/EBPβ in HepG2 cells treated with 10 ng/mL IL-6 for 15 min with and without 10 μM U0126 pretreatment. (**C**) PGRN protein expression in HepG2 cells treated with 10 ng/mL IL-6 for 6 hr with and without U0126 pretreatment. (**D**) Western blot assay of C/EBPβ protein level in HepG2 cells transfected with C/EBPβ siRNA (si-C/EBPβ) or negative control siRNA (si-NC). Ctr was parent HepG2 cells. (**E**) Intracellular PGRN protein expression in C/EBPβ-knockdown (si-C/EBPβ-1) and control HepG2 cells (si-NC) with and without IL-6 treatment for 6 hr. GAPDH was a loading control.

We next explored the molecular mechanism by which IL-6 regulates PGRN expression in HCC cells. Phosphorylation of Erk at Thr202/Tyr204 and C/EBPβ at Thr235 was increased in HepG2 cells with IL-6 treatment (Fig. 2A). In addition, IL-6-stimulated phosphorylation of C/EBPβ in HepG2 cells was markedly attenuated by U0126, an inhibitor of MEK/Erk signaling pathway (Fig. 2B), so the MEK/Erk pathway is required in IL-6-promoted activation of C/EBPβ. U0126 treatment inhibited IL-6-driven PGRN expression (Fig. 2C). To determine whether IL-6-driven PGRN expression depended on activation of C/EBPβ, we transfected HepG2 cells with specific C/EBPβ siRNA, which markedly inhibited endogenous C/EBPβ as compared with the control siRNA and parent HepG2 cells (Fig. 2D). The IL-6-stimulated expression of PGRN in HepG2 cells was lower with si-C/EBPβ than control siRNA (Fig. 2E).

### PGRN contributed to the oncogenic role of IL-6 in HepG2 cells.

**Figure 3:**
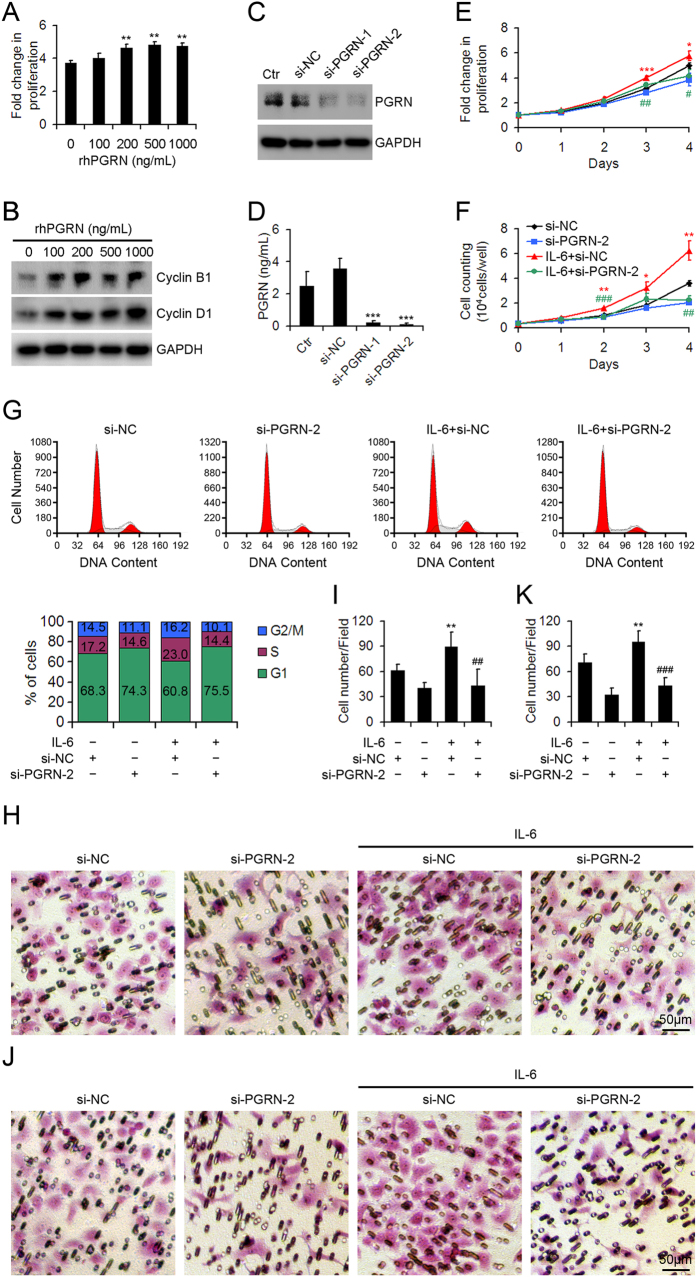
Inhibition of PGRN expression reduced IL-6-stimulated HepG2 cell proliferation, migration and invasion. (**A**) Cell proliferation of HepG2 cells treated with indicated doses of recombinant human PGRN (rhPGRN) at day 3. The relative absorbance was determined by setting the absorbance on day 0 to 1. Data are mean ± SD. **p < 0.01 compared with 0 ng/mL rhPGRN treatment. (**B**) Western blot assay of cyclin B1 and D1 protein levels in HepG2 cells treated with indicated doses of rhPGRN for 12 hr. (**C**) Western blot assay of PGRN protein level in HepG2 cells transfected with PGRN siRNA (si-PGRN) or negative control siRNA (si-NC). Ctr was parent HepG2 cells. GAPDH was a loading control. (**D**) ELISA assay of secreted PGRN from HepG2 cells transfected with PGRN siRNA (si-PGRN) or negative control siRNA (si-NC). Cell proliferation (**E**), cell counting (**F**) and cell cycle (**G**) assays in PGRN-knockdown (si-PGRN-2) and control HepG2 cells (si-NC) with and without 50 ng/mL IL-6. Representative micrographs showing the migration (**H**) and invasion (**J**) of PGRN-knockdown (si-PGRN-2) and control HepG2 cells (si-NC) with and without 50 ng/mL IL-6 assessed by transwell migration and invasion assays. Data analysis of migrated (**I**) and invaded (**K**) cells quantified by counting five fields under 20 × magnification. Data are mean ± SD. *p < 0.05; **p < 0.01; ***p < 0.001 compared with si-NC; ^#^P < 0.05; ^##^P < 0.01; ^###^P < 0.001 compared with IL-6 + si-NC.

Proliferative ability was dose-dependently increased in HepG2 cells with rhPGRN treatment (Fig. 3A). Furthermore, the expression of cyclin B1 and cyclin D1 was increased in HepG2 cells with 500-ng/mL rhPGRN treatment as compared with the control (Fig. 3B), which suggests that PGRN has a growth-promoting effect on HCC cells. To determine whether PGRN contributes to the oncogenic role of IL-6, we knocked down the expression of PGRN in IL-6-treated HepG2 cells and detected proliferation ability. Transfection of HepG2 cells with si-PGRN significantly inhibited the intracellular and secreted protein levels of PGRN (Fig. 3C,D). Knocking down PGRN expression decreased IL-6-stimulated proliferation of HepG2 cells (Fig. 3E,F). In addition, IL-6 stimulation enhanced the percentage of HepG2 cells in S and G2/M phases, which were impaired by reduced expression of PGRN (Fig. 3G). Furthermore, knocking down PGRN expression decreased IL-6-stimulated migration and invasion of HepG2 cells (Fig. 3H–K).

### PGRN activated mTOR signaling in HepG2 cells.

**Figure 4:**
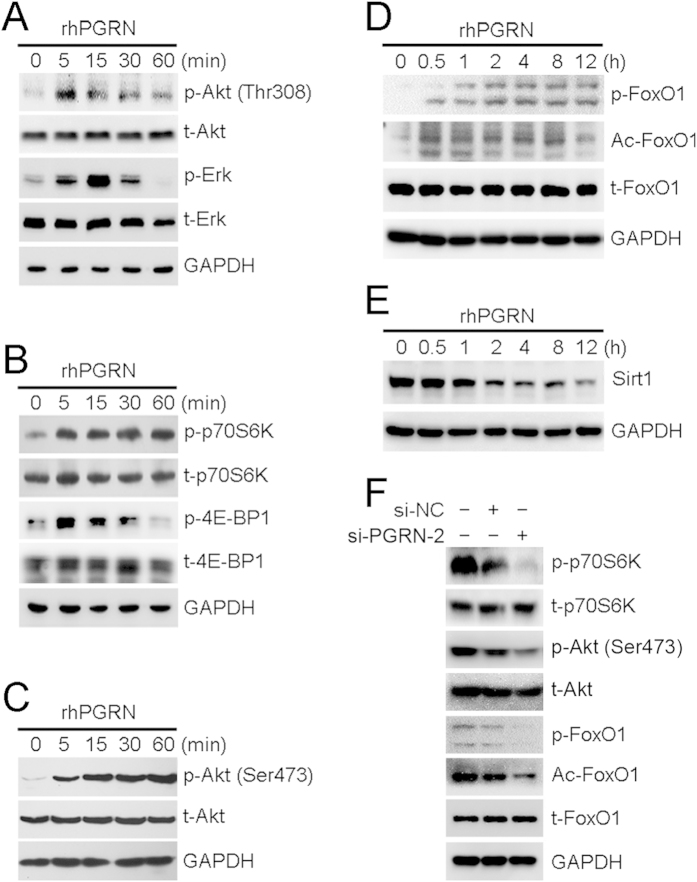
rhPGRN treatment promoted the activation of mammalian target of rapamycin (mTOR) signaling. (**A**) Western blot assay of Akt and Erk activation with 500 ng/mL rhPGRN treatment in HepG2 cells. Phosphorylation of mTORC1 downstream molecules (**B**) and mTORC2-dependent Akt at Ser473 (**C**) in HepG2 cells with rhPGRN treatment. Phosphorylation and acetylation of FoxO1 (**D**) and Sirt1 protein expression (**E**) in HepG2 cells with rhPGRN treatment. (**F**) Western blot assay of mTOR signaling in HepG2 cells transfected with PGRN siRNA (si-PGRN-2) or negative control siRNA (si-NC). GAPDH was a loading control.

To elucidate the molecular mechanism by which PGRN upregulates the proliferation of HCC cells, we detected the activation of Akt, Erk and mTOR signaling in HepG2 cells. Phosphorylation of Akt at Thr308 and Erk at Thr202/Tyr204 was increased in HepG2 cells with 500 ng/mL rhPGRN treatment (Fig. 4A). For mTOR signaling, HepG2 cells treated with rhPGRN showed increased phosphorylation of translational regulators 4E-BP1 and p70S6K, the main downstream targets of mTORC1 (Fig. 4B); increased phosphorylation of Akt at Ser473, mTORC2-dependent Akt phosphorylation (Fig. 4C); increased Akt-specific phosphorylation of FoxO1 at Ser256 (Fig. 4D); and induced acetylation of FoxO1 along with decreased expression of NAD-dependent deacetylase Sirt1 (Fig. 4D,E). In addition, the phosphorylation of p70S6K and Akt at Ser473, and phosphorylation and acetylation of FoxO1 were lower in HCC cells with PGRN siRNA than control siRNA and parent HepG2 cells (Fig. 4F).

### Inhibition of mTOR signaling attenuated the proliferation and survival-promoting effects of PGRN in HepG2 cells.

**Figure 5:**
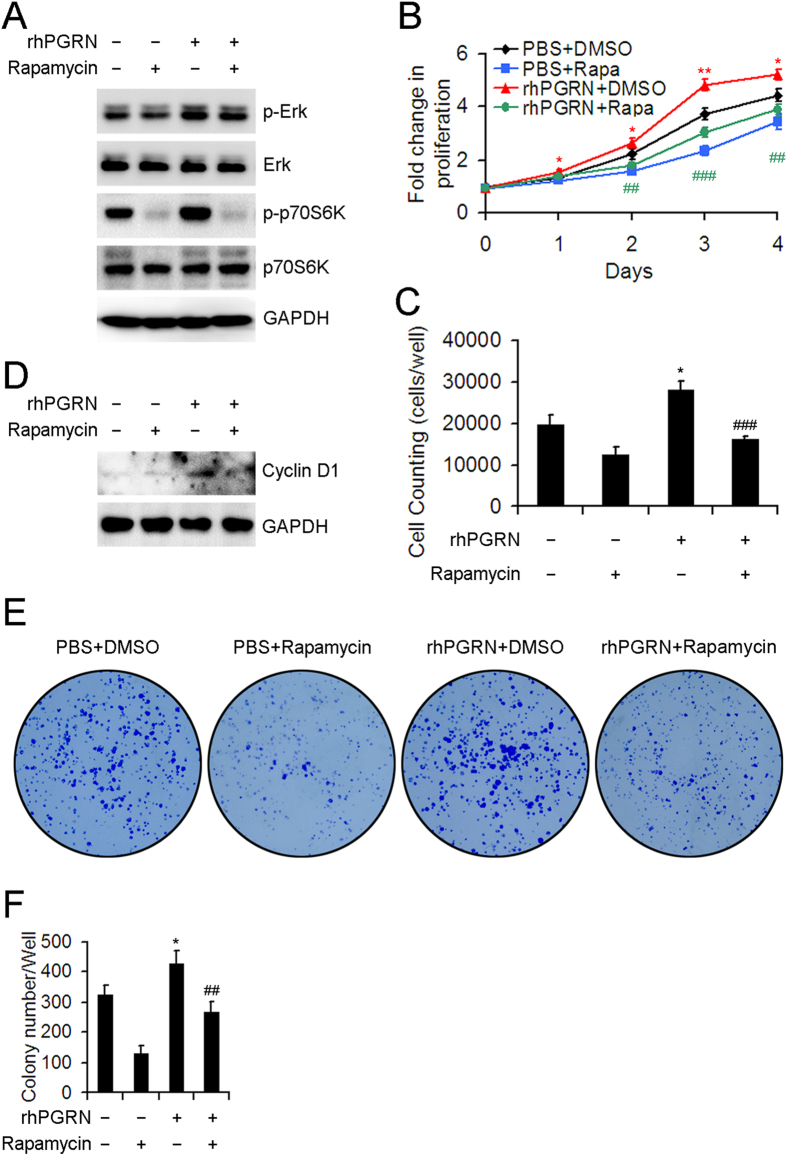
Rapamycin pretreatment inhibited PGRN-stimulated proliferation and survival of HepG2 cells. (**A**) Western blot assay of the phosphorylation of Erk and p70S6K with 500-ng/mL rhPGRN treatment of HepG2 cells for 15 min with and without 100 μM rapamycin pretreatment. Cell proliferation (**B**) and cell counting (**C**) assays of rhPGRN-treated HepG2 cells with and without rapamycin pretreatment. (**D**) Protein level of cyclin D1 in HepG2 cells with rhPGRN treatment in the presence and absence of rapamycin. (**E**) Representative micrographs showing colony formation of rhPGRN-treated HepG2 cells with and without rapamycin pretreatment in 1% fetal bovine serum. (**F**) Data analysis of colony numbers. Data are mean ± SD. *P < 0.05, **P < 0.01 compared with PBS + DMSO. ^##^P < 0.01, ^###^P < 0.001 compared with rhPGRN + DMSO.

We next determined the role of mTOR signaling in PGRN-regulated proliferation of HCC cells. Pretreatment with 100 nM rapamycin, a specific mTOR inhibitor, blocked PGRN-induced phosphorylation of p70S6K but not Erk in HepG2 cells (Fig. 5A). Inhibition of mTOR signaling disturbed PGRN-stimulated proliferation of HepG2 cells (Fig. 5B,C). In addition, rapamycin pretreatment effectively attenuated the increased protein level of cyclin D1 in HepG2 cells with rhPGRN treatment (Fig. 5D). We further detected the role of PGRN in survival of HCC cells under low serum conditions. Treatment with rhPGRN effectively ameliorated the decreased colony number induced by 1% FBS treatment, which was reversed with rapamycin pretreatment (Fig. 5E,F). These data indicated that the regulatory role of PGRN in the proliferation and survival of HCC cells at least in part depends on the activation of mTOR signaling.

### Rapamycin inhibited the migration and invasion of HepG2 cells with rhPGRN administration.

**Figure 6:**
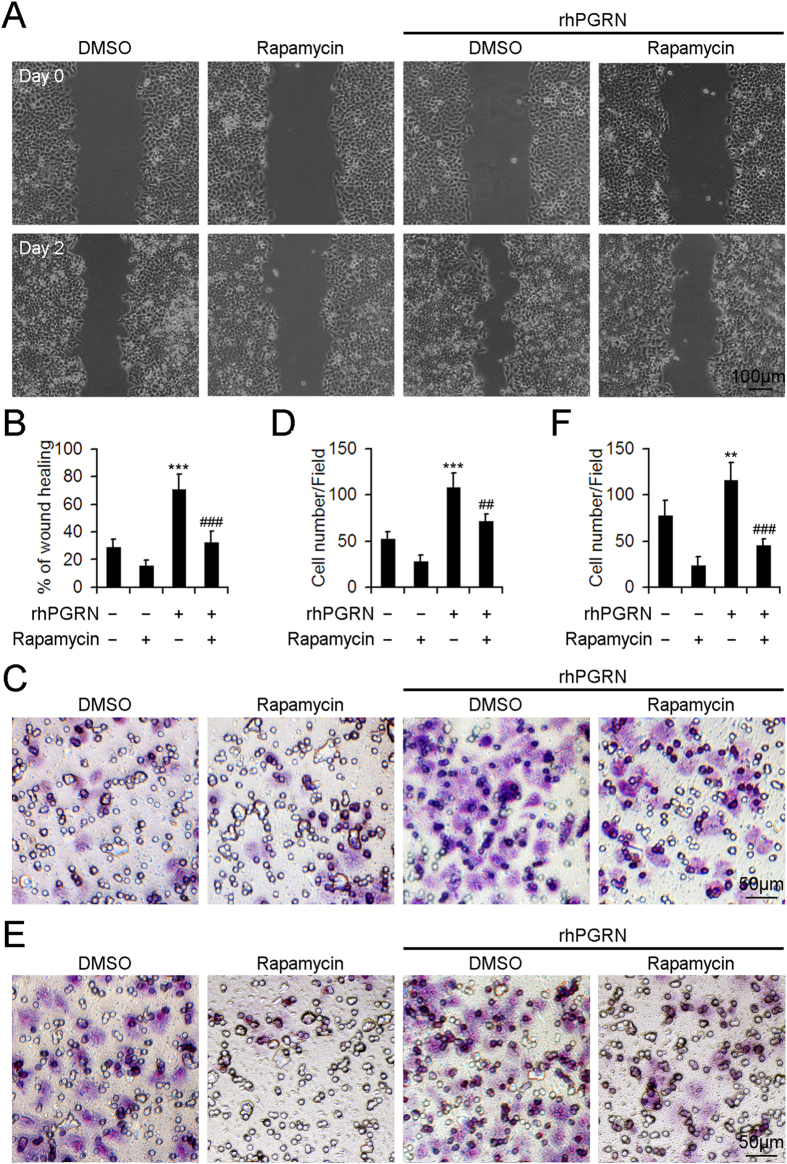
Inhibition of mTOR signaling interfered with PGRN-induced migration and invasion of HepG2 cells. (**A**) Representative micrographs showing the wound closure of monolayer HepG2 cells with phosphate buffered saline (PBS) or rhPGRN treatment with and without rapamycin pretreatment. (**B**) The percentage of wound healing. The normalized values were calibrated against the wound widths at day 0 that were arbitrarily set to 100%. Representative micrographs showing the migration (**C**) and invasion (**E**) of HepG2 cells with PBS or rhPGRN treatment with and without rapamycin pretreatment assessed by transwell migration and invasion assays. Data analysis of migrated (**D**) and invaded (**F**) cells quantified by counting five fields under 20 × magnification. Data are mean ± SD. **P < 0.01, ***P < 0.001 compared with DMSO. ^##^P < 0.01, ^###^P < 0.001 compared with rhPGRN + DMSO.

Besides a growth-promoting effect, PGRN also contributes to other behaviors associated with its stimulatory role in tumorigenesis such as increased cell migration and invasion. We examined the effect of rapamycin on the increased motility of cells in response to rhPGRN by monolayer wound assay (Fig. 6A). After 2 days, HepG2 cells treated with rhPGRN in the absence of rapamycin showed enhanced wound closure as compared with cells without rhPGRN treatment (Fig. 6B). Exposure to rapamycin along with rhPGRN reduced the wound closure of HepG2 cells from 71.2% to 32.4% (Fig. 6B). These results were confirmed by transwell migration assay (Fig. 6C). Rapamycin disturbed the enhanced migration of HepG2 cells induced by rhPGRN (Fig. 6D). We next tested the effect of inhibiting mTOR signaling on the invasive behavior of HepG2 cells by *in vitro* invasion assay (Fig. 6E). Invasive behavior was greater for HepG2 cells with than without rhPGRN treatment. In contrast, the addition of rapamycin with rhPGRN reduced the invasion ability as compared with rhPGRN alone (Fig. 6F). Activation of mTOR signaling in response to PGRN plays an essential role in the increased motility, migration and invasion of HCC cells.

### PGRN-mediated mTOR signaling contributed to IL-6-stimulated proliferation, migration and invasion of HCC cells.

**Figure 7:**
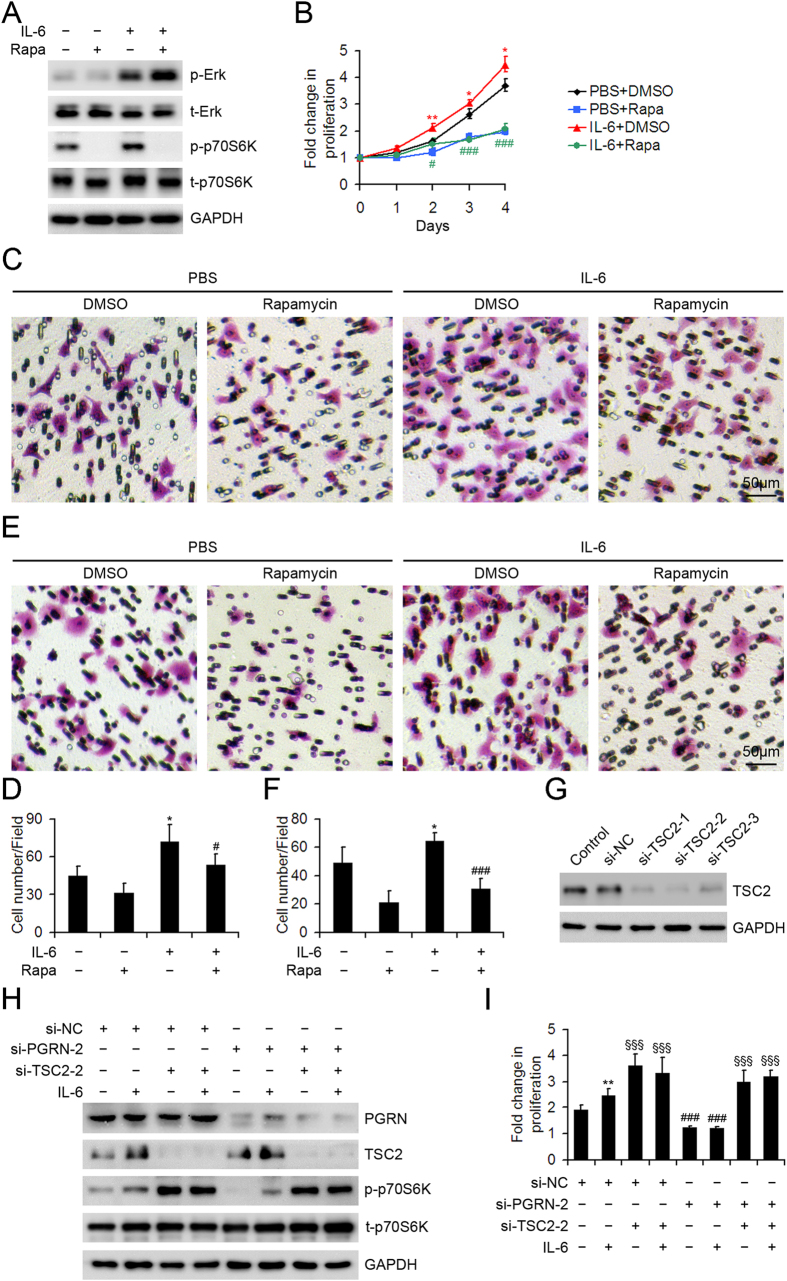
PGRN-mediated mTOR signaling contributed to IL-6-stimulated behaviors of HepG2 cells. (**A**) Western blot assay of the phosphorylation of Erk and p70S6K with 10-ng/mL IL-6 treatment of HepG2 cells for 30 min with and without 100 μM rapamycin pretreatment. GAPDH was a loading control. (**B**) Cell proliferation assay of IL-6-treated HepG2 cells with and without rapamycin pretreatment. Representative micrographs showing the migration (**C**) and invasion (**E**) of HepG2 cells with PBS or IL-6 treatment with and without rapamycin pretreatment assessed by transwell migration and invasion assays. Data analysis of migrated (**D**) and invaded (**F**) cells quantified by counting five fields under 20 × magnification. Data are mean ± SD. *P < 0.05, **P < 0.01 compared with PBS + DMSO. ^#^P < 0.05, ^###^P < 0.001 compared with IL-6 + DMSO. (**G**) Western blot assay of TSC2 protein level in HepG2 cells transfected with TSC2 siRNA (si-TSC2) or negative control siRNA (si-NC). Ctr was parent HepG2 cells. (**H**) Western blot assay of PGRN, TSC2, phospho-p70S6K and p70S6K in HepG2 cells transfected with indicated siRNA with or without IL-6 treatment for 30 min. GAPDH was a loading control. (**I**) Cell proliferation assay of HepG2 cells transfected with indicated siRNA with or without IL-6 treatment for 3 days. **P < 0.01, compared with HepG2 cells without IL-6 treatment. ^###^P < 0.001 compared with HepG2 cells without si-PGRN-2 transfection. **^§^**P < 0.05, **^§§§^**P < 0.001 compared with HepG2 cells without si-TSC2-2 transfection.

To explore the connection between mTOR signaling and IL-6 in HCC development, we investigated the behaviors of IL-6-treated HepG2 cells with or without mTOR signaling inhibition. IL-6 treatment enhanced the levels of phospho-Erk and p70S6K, and only IL-6-stimulated mTOR signaling was blocked by rapamycin pretreatment (Fig. 7A). Inhibition of mTOR signaling by rapamycin effectively diminished IL-6-stimulated proliferation, migration and invasion of HepG2 cells (Fig. 7B–F). To determine whether PGRN-mediated mTOR signaling is involved in the oncogenic role IL-6 in HCC, a rescue study was performed by persistent activation of mTOR signaling in IL-6-treated HepG2 cells transfected with control or specific PGRN siRNA. We knocked down the expression of tuberous sclerosis complex 2 (TSC2), the key negative regulator of mTOR signaling, in HepG2 cells (Fig. 7G). TSC2 knocking down resulted in marked activation of mTOR signaling, evidenced by substantially elevated phospho-p70S6K levels in HepG2 cells regardless of PGRN knocking down (Fig. 7H). The proliferation assay revealed that PGRN knocking down attenuated IL-6-stimulated proliferation in HepG2 cells, which was restored by si-TSC2-mediated rescue of mTOR signaling (Fig. 7I).

### Rapamycin attenuated PGRN-stimulated growth of tumors *in vivo*.

**Figure 8:**
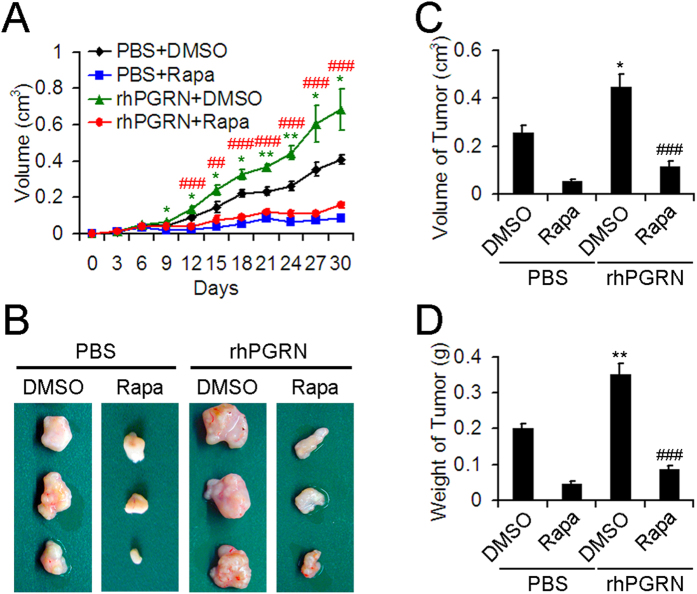
Rapamycin reduced PGRN-stimulated HCC tumor growth in nude mouse xenografts. Tumor growth curves (**A**) and photographs (**B**) of tumors with HepG2 cell implantation and treated with PBS or rhPGRN concomitant with DMSO or rapamycin. Volume (**C**) and weight (**D**) of tumors from mice with HepG2 cell implantation and indicated treatment. Data are mean ± SEM. *P < 0.05, **P < 0.01 compared with PBS + DMSO. ^##^P < 0.01, ^###^P < 0.001 compared with rhPGRN + DMSO.

To investigate whether PGRN-stimulated mTOR signaling could be a therapeutic target in HCC, we subcutaneously injected nude mice with HepG2 cells administered with or without rhPGRN in the absence or presence of rapamycin. Tumor growth was substantially elevated with rhPGRN administration as compared with phosphate buffered saline (PBS) treatment (Fig. 8A,B). Tumor volume and weight were greater in mice with than without rhPGRN treatment (Fig. 8C,D). As well, the growth-promoting role of PGRN in tumors from mice with transplantation of HepG2 cells was inhibited by rapamycin treatment (Fig. 8A–D). Thus, PGRN-stimulated mTOR signaling is crucial for HCC and may be a promising therapeutic target.

## Discussion

Despite the recent advances in diagnosis and therapy of HCC, the cancer remains one of the leading causes of cancer-related death and severely affects public health worldwide^29^. Liver transplantation or surgical resection is the first-line treatment for HCC^30^. Most HCC patients present at advanced stages and cancer is inoperable; chemotherapy is second-line treatment, but the overall response rate is unsatisfactory^31^. Molecule-targeted therapy based on the study of HCC carcinogenic mechanisms is a new approach in liver cancer treatment. The multi-kinase inhibitor sorafenib, a molecule-targeted therapeutic drug is the only approved drug for HCC^32^. However, the treatment has not been entirely satisfactory^33^,^34^. Therefore, further understanding of the molecular mechanisms of hepatocarcinogenesis and novel therapies are urgently needed.

In the present study, we aimed to examine the potential role of the IL-6/PGRN/mTOR pathway in the carcinogenesis of HCC. We found that the expression of IL-6 and PGRN were increased and positively correlated in HCC tissues. Consistent with our results that IL-6 treatment enhanced the expression and secretion of PGRN in HCC cell lines, increased PGRN level has been found in cholangiocarcinama cells with IL-6 treatment^22^. Besides HCC cell lines, other cell types were tested in present study and IL-6 treatment also enhanced PGRN expression in lung, cervical and breast cancer cell lines, suggesting IL-6-driven PGRN expression is a common phenomenon. Here we also found that the elevated expression of IL-6 and PGRN in HCC mainly presented in different physiological tissues, which suggests that mesenchymal cells-produced IL-6 promotes PGRN expression in epithelial cells through a paracrine mode during HCC development. HCC is an inflammation-related cancer and often develops in association with chronic liver inflammation caused by HBV or HCV infection^35^,^36^. Previous studies suggested that IL-6 level is increased in many cancers, including liver cancer^37^, and serum IL-6 levels are associated with poor prognosis in HCC^38^,^39^,^40^,^41^,^42^. That PGRN is highly expressed in HCC as well implies a potential association of IL-6 and PGRN as non-cellular components of the inflammatory microenvironment in HCC.

IL-6 can activate Janus kinase (JAK)-signal transducer and activator of transcription 3 (STAT3), MEK-Erk and PI3K/Akt signaling via its unique receptor and the common signaling receptor subunit glycoprotein130 (gp130)^43^,^44^. In our study, treatment with the inhibitor of MEK/Erk signaling attenuated IL-6–induced PGRN expression in HepG2 cells. Human C/EBPβ is activated by Erk-dependent phosphorylation of Thr-235^45^,^46^. In addition, putative binding sites for C/EBPβ have been identified in the 5′ sequences of the genes for human and mouse *grn*^47^. Here we showed that inhibiting MEK/Erk signaling decreased IL-6–stimulated activation of C/EBPβ, and the reduced C/EBPβ expression led to reduced expression of PGRN in HepG2 cells induced by IL-6. These data demonstrate that IL-6 stimulates the expression of PGRN in HCC cells mediated by IL-6-activated MEK/Erk/C/EBPβ signaling. In addition, reduced expression of PGRN decreased IL-6–stimulated proliferation, migration and invasion of HepG2 cells, which suggests that PGRN may contribute to the IL-6–mediated malignancy of HCC cells, at least in part.

In light of the effect of PGRN on the behavioral change of HCC cells, we investigated the molecular mechanisms of hepatocarcinogenesis regulated by PGRN. PGRN-supported tumor growth requires the activation of typical growth factor signaling, including MEK/Erk and PI3K/Akt signaling^17^. Here we observed enhanced phosphorylation of Akt at Thr308 and Erk1/2 in HCC cells stimulated with rhPGRN, for activated PI3K/Akt and MEK/Erk signaling. The phosphorylation of p70S6K is increased in R-cells (a mouse embryos-derived 3T3-like cell) with PGRN overexpression^48^ and in mouse myoblast C2C12 cells treated with mouse PGRN^18^. In our study, rhPGRN-treated HepG2 cells showed enhanced phosphorylation of mTORC1 downstream targets p70S6K, 4E-BP1 and mTORC2-dependent Akt Ser473, which provides the first evidence for PGRN-stimulated activation of mTOR signaling in human cancer cells.

The oncogenic role of PGRN in cancer cells was recently linked to a member from the forkhead transcription factor family, FoxO1^22^,^29^,^49^, a target of mTORC2-regulated Akt^50^,^51^. In the present study, we demonstrated that PGRN enhanced the phosphorylation and acetylation of FoxO1 and inhibited the expression of Sirt1 in HCC cells, which is consistent with our current knowledge of how FoxO1 is regulated. Akt mediates the phosphorylation of FoxO1, which leads to its export from the nucleus and inhibiting its transcriptional activity^52^; deacetylase Sirt1 mediates the deacetylation of FoxO1, thereby leading to its nuclear retention and accumulated transcriptional activity^53^,^54^.

The role of PGRN in cell proliferation, migration, and survival is well established in various experimental models of cell lines and animals^17^. We found that rhPGRN treatment enhanced the proliferation of HepG2 cells *in vitro* and tumorigenic potential in athymic nude mice. Furthermore, rhPGRN contributed to survival of HepG2 cells under low serum conditions. In line with the findings that reduced PGRN mRNA expression decreases HCC cell invasion^20^, we found that rhPGRN treatment enhanced the migration and invasive behavior of HepG2 cells in *in vitro* assays. Importantly, inhibition of mTOR signaling by rapamycin pretreatment effectively attenuated PGRN- and IL-6-mediated oncogenic behaviors in HepG2 cells, respectively. In addition, the proliferation-promoting role of IL-6 attenuated by lowered PGRN expression was restored by persistent activation of mTOR signaling in HCC cells. These data strongly suggest a crucial contribution of IL-6/PGRN/mTOR signaling in hepatocarcinogenesis.

Preclinical *in vitro* and *in vivo* studies have shown reduced HCC tumor growth with mTOR inhibitors^55^. Rapamycin alone or in combination with other therapeutic agents targeting the epidermal growth factor receptor (EGFR) or vascular endothelial growth factor (VEGF) prolonged the survival of HepG2 cells-xenografted mice^57^,^58^, suggesting a possible clinical benefit of the therapeutic strategies targeting mTOR signaling. Currently, the first-generation mTOR inhibitors (rapalogs) are used in clinical trials for treating HCC but do not show the expected efficacy; combining mTOR inhibition with sorafenib enhanced the antitumoral effect in cancer models, including HCC^56^. Our study indicates the IL-6–drived PGRN and PGRN-activated mTOR signaling may be a molecular target for treatment of HCC.

Collectively, our data show that PGRN is an IL-6–response gene and contributes to the role of IL-6 in HCC cell proliferation. In addition, PGRN-mediated malignancy of HCC depends on PGRN-stimulated mTOR signaling. These findings provide new insights into the oncogenic activity of PGRN in hepatocarcinogenesis *in vitro* and *in vivo* but may also reveal the IL-6/PGRN/mTOR cascade as a novel target in the treatment of HCC.

## Methods

### Hepatocellular carcinoma tissue array analysis.

To detect the expression of IL-6 and PGRN in normal and HCC tissue, the human liver carcinoma and normal tissue microarrays (BC03119a, US Biomax, Inc., Rockville, MD) containing 95 HCC and 10 normal liver tissues were examined by immunohistochemical staining. This part of the study did not require approval from the Institutional Review Board as the tissue microarray was commercially sourced. Immunohistochemistry was performed as described^59^ with IL-6 antibody (1:400, Abcam, Cambridge, UK) and PGRN antibody (1:50, Santa Cruz Biotechnology, Dallas, TX). Semi-quantitative analysis of the staining intensity score was as described^22^.

### Cell culture, treatment and transfection.

HepG2, Bel-7402, A549, HeLa and MCF-7 cell lines were procured from the American Type Culture Collection (Manassas, VA). All cell lines were maintained in high glucose Dulbecco’s modified Eagle’s medium (DMEM; Invitrogen, Carlsbad, CA) with 10% fetal bovine serum (FBS; Invitrogen), 1% penicillin/streptomycin (Sigma-Aldrich, St. Louis, MO, USA) and 2 mmol/L L-glutamine at 37 °C with 5% CO_2_ in a humidified incubator. Upon reaching 80-90% confluence, HCC cells were treated with 0, 5, 10, 20 or 50 ng/mL recombinant human IL-6 (Peprotech, Rocky Hill, NJ) or 0, 100, 200, 500, 1000 ng/mL recombinant human PGRN (rhPGRN; generated and purified as reported^60^) for the indicated times. For signaling pathway analysis, HepG2 cells were stimulated with 10 ng/mL IL-6 or 500 ng/mL rhPGRN for the indicated times after serum-free treatment for 18 h. For inhibitor treatment, cells were replaced with dimethyl sulfoxide (DMSO; Sigma-Aldrich), U0126 (10 μM; Cell Signaling Technology, Danvers, MA) or rapamycin (100 μM; Sigma-Aldrich) containing medium for 1 hr before 10 ng/mL IL-6 or 500 ng/mL rhPGRN. For siRNA transfection, HepG2 cells were seeded into 6-well culture plates at 1 × 10^5^ cells/well and cultured in medium without antibiotics. siRNA oligonucleotides (RiboBio, Guangzhou, China) were transfected into cells by the Lipofectamine 2000 reagent method (Invitrogen). The siRNA target sequences for human *GRN* were 5′-CTGTGTGTGACCTGATCCA-3′ and 5′-GTGAGCTGCCCAGATGGCT-3′, for human *CEBPB* 5′-CCAAGAAGACCGTGGACAA-3′, 5′-GGGGCAAGAACTGCAAGAA-3′, and 5′-TGCGGAACTTGTTCAAGCA-3′, and for human TSC2 5′-GCATTAATCTCTTACCATA-3′, 5′-GGGACATTCTGCTGAACAT-3′ and 5′-GGATTACCCTTCCAACGAA-3′. Cells were harvested and used for experiments 48 hr after transfection.

### RNA extraction and real time RT-PCR.

Total RNA was isolated from cells by use of TRIzol reagent according to the manufacurer’s instructions (Life Technologies, Carlsbad, CA). Then 1 μg of DNA-free total RNA was reverse transcribed by use of a one-step RT-PCR kit (TaKaRa Bio, Shiga, Japan). Sequence-specific primers were synthesized for human PGRN, 5′-GGACAGTACTGAAGACTCTG-3′ and 5′-GGATGGCAGCTTGTAATGTG-3′; and human β-actin, 5′-CATGTACGTTGCTATCCAGGC-3′ and 5′-CTCCTTAATGTCACGCACGAT-3′. Reactions were performed in a 50-μL SYBR GREEN PCR volume in a 96-well optical reaction plate formatted in the Bio-Rad iCycler system (Bio-Rad, Hercules, CA). β-actin was an internal control for RNA quality and differences among samples.

### Western blot assay.

Total protein was extracted from cells by the use of NP-40 lysis buffer (Beyotime Institute of Biotechnology, China). Western blot assay was performed as described^59^ with primary antibodies for PGRN (1:1000), total-C/EBPβ (1:1000), acetylated-FoxO1 (1:2000, Santa Cruz Biotechnology), cyclin B1 (1:2000), cyclin D1 (1:2000), sirtuin 1 (Sirt1; 1:1000, ProteinTech Group, Chicago, IL), phospho-C/EBPβ (Thr235, 1:1000), phospho-Akt (Thr308, 1:1000), phospho-Akt (Ser473, 1:2000), total Akt (1:2000), phospho-Erk1/2 (Thr202/Tyr204, 1:2000) and total Erk1/2 (1:1000), phospho-p70S6K (Thr389, 1:1000), total p70S6K (1:1000), phospho-4E-binding protein 1 (phospho-4E-BP1, Thr37/46, 1:1000), total 4E-BP1 (1:1000), phospho-FoxO1 (Ser256, 1:1000), total-FoxO1 (1:1000) and TSC2 (1:1000, Cell Signaling Technology). GAPDH antibody was a control (1:2000, Hangzhou Goodhere Biotech, China).

### ELISA of PGRN secretion.

HepG2 cells were seeded into 6-well culture plates at 4 × 10^5^ cells/well; conditioned medium was collected 6 hr after 0, 5, 10, 20 or 50 ng/mL IL-6 stimulation. The amount of PGRN in conditioned medium was assayed by use of an ELISA kit (R&D Systems, Minneapolis, MN).

### Cell proliferation, cell cycle and colony formation assays *in vitro*.

HepG2 cells were seeded 2 × 10^3^ per well in 96-well plates. At days 0-4, cell proliferation was accessed by use of the Cell Counting Kit-8 (CCK-8; Dojindo Laboratories, Tokyo) and cell number counting assay as described^58^. For cell cycle analysis, HepG2 cells were seeded 1 × 10^5^ per well in 6-well plates with the indicated treatment for 48 hr, cells were collected and stained by use of a cell cycle kit (Shanhai BestBio, China). Cells were then assayed by flow cytometry (Beckman Coulter, Germany). For colony formation assay, HepG2 cells were seeded 1 × 10^4^ per well in 6-well plates in medium containing 1% FBS with the indicated treatment and incubated for 2 weeks, then clones were counted after fixing and staining with 0.5% crystal violet solution.

### Migration and invasion assays.

HepG2 cell monolayers were wounded with use of a plastic tip and treated with DMSO or 100 μM rapamycin 1 hr before PBS or 500 ng/mL rhPGRN treatment. The motility of cells was photographed under a light microscope at days 0, 1 and 2. In the transwell assay, HepG2 cells were pretreated with DMSO, 100 μM rapamycin 1 hr before PBS or 500 ng/mL rhPGRN treatment for 12 hr in DMEM containing 1% FBS. For transwell migration assay, 2 × 10^4^ cells suspended in DMEM without FBS were added to transwell inserts (8-μm pore size; Millipore, Billerica, MA), held in 24-well companion plates with DMEM containing 10% FBS, and incubated 24 hr. For transwell invasion assay, 2 × 10^4^ cells suspended in DMEM without FBS were added to ECMatrix-coated inserts (Millipore, Billerica, MA), held in 24-well companion plates with DMEM containing 10% FBS, and incubated for 48 hr. The cells and Matrigel in the upper chambers were removed by using a cotton tip. Migrating and invading cells at the bottom of the filter were photographed under a light microscope after fixing and visualizing by Giemsa staining. The number of migrating and invading cells in each chamber was quantified by counting five fields under 20 × magnification.

### Xenograft tumor studies.

The experimental protocols were approved by the Institutional Animal Care and Use Committee of Shandong University [Permit Number: KYLL-2013-112]. The investigation conformed to the US National Institutes of Health Guide for the Care and Use of Laboratory Animals and was performed in accordance with the ARRIVE guidelines (http://www.nc3rs.org/ARRIVE). All mice were housed under specific pathogen-free conditions and maintained on a 12-hr light/dark cycle at 25 ± 2 °C, with free access to food and water. Six-week-old female BALB/c nude mice (nu/nu) were purchased from Vital River Laboratories (Bejing) and acclimated to housing conditions for at least 1 week before experiments.

We subcutaneously injected mice with 110^7^ HepG2 cells, then on days 5 and 6 after injection, divided mice into 4 groups (n = 8 each) for intraperitoneal injection of rhPGRN (10 mg/kg body weight) or PBS and rapamycin (1.5 mg/kg body weight) or DMSO every 3 days. Tumor size was measured every 3 days by the use of calipers and calculated as V (mm^3^) = 0.5 × ab^2^, where a and b represent the long diameter and perpendicular short diameter (mm) of the tumor, respectively. At the end of experiments, mice were killed by cervical dislocation under sodium pentobarbital (50 mg/kg) anesthesia and tumors were excised and weighed.

### Statistical analysis.

Data are expressed as mean ± SD or SEM. Differences were estimated by one-way ANOVA followed by Duncan’s multiple range test. *P *< 0.05 was considered statistically significant. Correlation of IL-6 expression with PGRN expression in HCC tissues was analyzed by Pearson correlation test.

## Additional Information

**How to cite this article**: Liu, F. *et al.* Interleukin-6-stimulated progranulin expression contributes to the malignancy of hepatocellular carcinoma cells by activating mTOR signaling. *Sci. Rep.* **6**, 21260; doi: 10.1038/srep21260 (2016).
